# Supplementary material for: Cascadia low frequency earthquakes at the base of an overpressured subduction shear zone
Source: Nat Commun. 2020 Aug 3;11:3874. doi: 10.1038/s41467-020-17609-3 (PMC7398906; doi:10.1038/s41467-020-17609-3)
Supplement: Supplementary file 1 — Supplementary Information [file 41467_2020_17609_MOESM1_ESM.pdf]

SUPPLEMENTARY INFORMATION

Cascadia low frequency earthquakes at the base of an overpressured subduction shear zone

Andrew J. Calvert[1], Michael G. Bostock[2], Geneviève Savard[3], and Martyn J. Unsworth[4]

[1]*Department of Earth Sciences, Simon Fraser University, 8888 University Drive, Burnaby, British Columbia, V5A 1S6, Canada*

[2]*Department of Earth, Ocean and Atmospheric Sciences, 2207 Main Mall, University of British Columbia, British Columbia, V6T 1Z4, Canada*

[3]*Department of Geosciences, University of Calgary, 2500 University Drive NW, Calgary, Alberta, T2N 1N4, Canada*

[4]*Department of Physics, University of Alberta, Edmonton, Alberta, T6G 2E9, Canada*

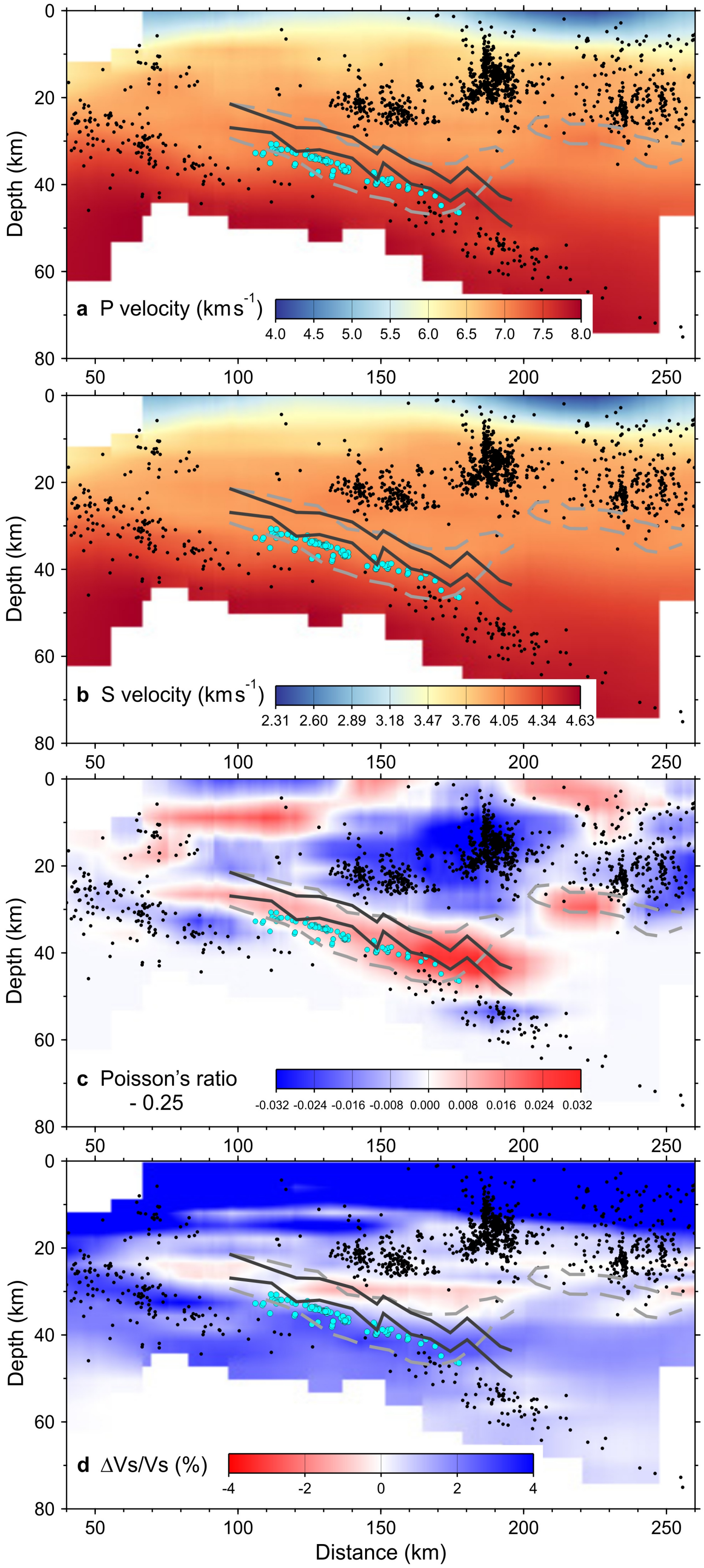

Supplementary Fig. 1: Velocity models along the POLARIS teleseismic profile. **a**, P wave velocity: locally elevated values are visible at 30 km depth at a distance of 210-240 km and at the ends of the landward dipping region of high Poisson's ratio inferred from the relative travel times of teleseismic phases (solid grey lines). **b**, S wave velocity, showing a relatively smooth variation along the profile. The colour scale is that used in **a** scaled by 0.577, which corresponds to a Poisson's ratio of 0.25. **c**, Deviation in Poisson's ratio from 0.25, which exhibits a similar variation to $(\lambda-\mu)/\rho$ (Fig. 2c), and indicates that elevated values correspond to the negative S velocity perturbation inferred from teleseismic migration (grey dashed line), which is shown in Fig. 2d. **d**, Downward S velocity perturbation calculated from tomographic S velocity model. Distance scale as in ref. 1. Vertical exaggeration is 1.5.

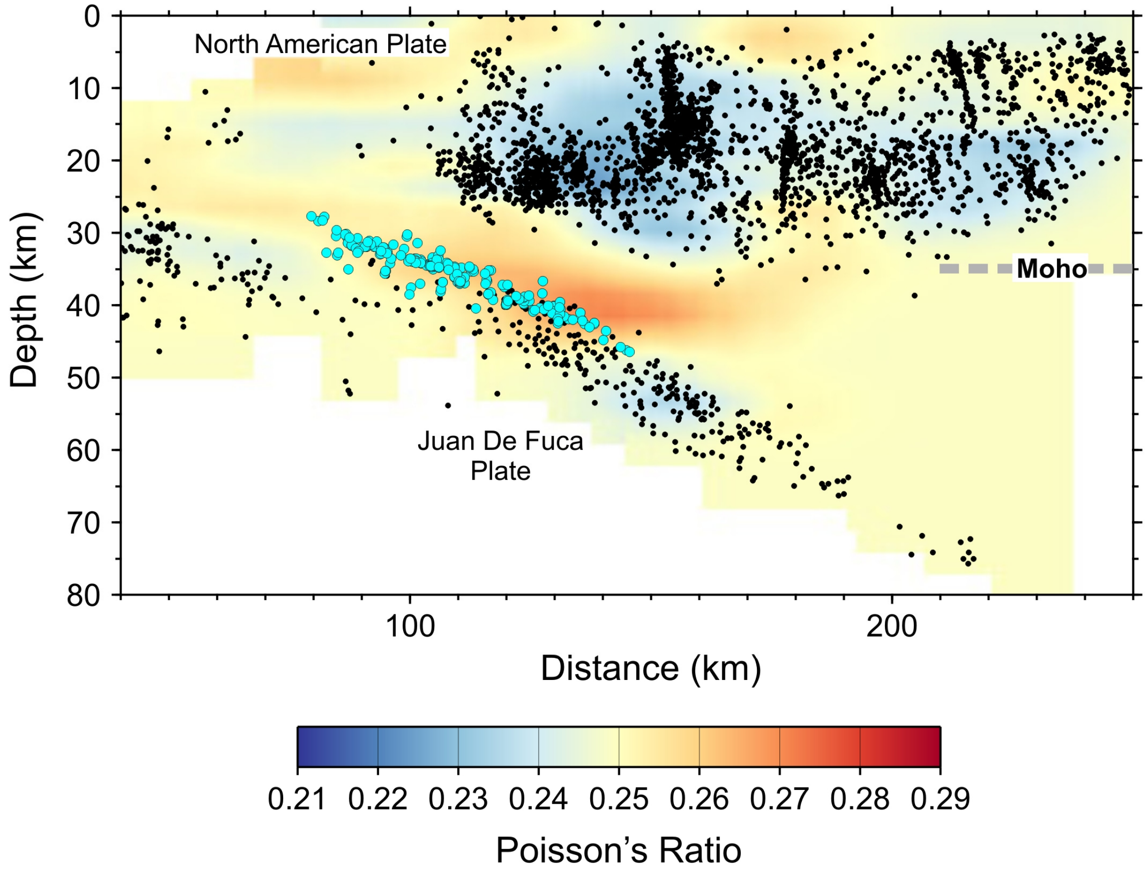

Supplementary Fig. 2: Poisson's ratio sections along dip profile XX'. The values of Poisson's ratio are averaged over 100 km along strike, and show how the LFEs (filled blue circles) project into the top of the inslab seismicity. The LFEs and conventional seismicity (filled black circles) occur in different volumes, and the apparent overlap at 38-45 km depth is due to the 2D representation of the 3D distribution. The continental Moho is indicated at approximately 35 km depth. The location of profile XX' is shown in Supplementary Note 1.

Supplementary Note 1: Resolution tests for 3-D tomographic velocity model

The 3D P wave and S wave velocity models and the below resolution tests have been published previously[1]. The resolution tests are reproduced here to permit evaluation of the velocity models presented in the main text; the POLARIS (Portable Observatories for Lithospheric Analysis and Research Investigating Seismicity) profile corresponds to profile BB'.

First, we performed a checkerboard resolution test with a +/- 5% anomaly in P-wave velocity on 2x2x2 grid cells, which correspond to cells of 24 x 24 x 6 km^3. We extracted results for sections AA', BB' and CC', as located in Supplementary Fig. 3, and display them in Supplementary Figs. 4 and 5.

Second, we inverted synthetic travel times from a model comprising a background 1D P wave velocity variation with a superimposed dipping layer 8 km thick with a *V*p/*V*s ratio of 2.35 approximately following published slab interface models[2,3]. Supplementary Figs. 6 and 7 display our results for sections AA', BB' and CC'.

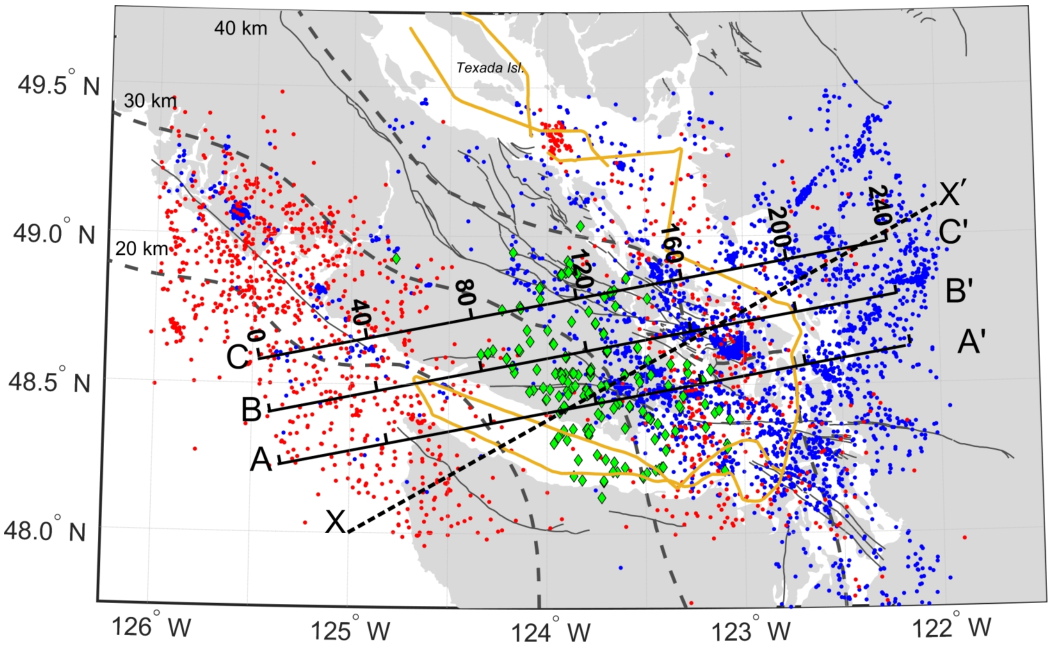

Supplementary Fig. 3: Relocated epicentres after inversion of the full traveltime data set. Red dots are intraslab earthquakes located below the plate interface model of ref. 2, blue dots are crustal events located above, and green diamonds are LFEs. Positions of profiles AA', BB', CC' , and XX' are indicated. Solid black lines mark known forearc faults and thick dashed grey lines are the plate contours at 20, 30 and 40 km from ref. 1. Yellow lines are SHIPS seismic profiles.

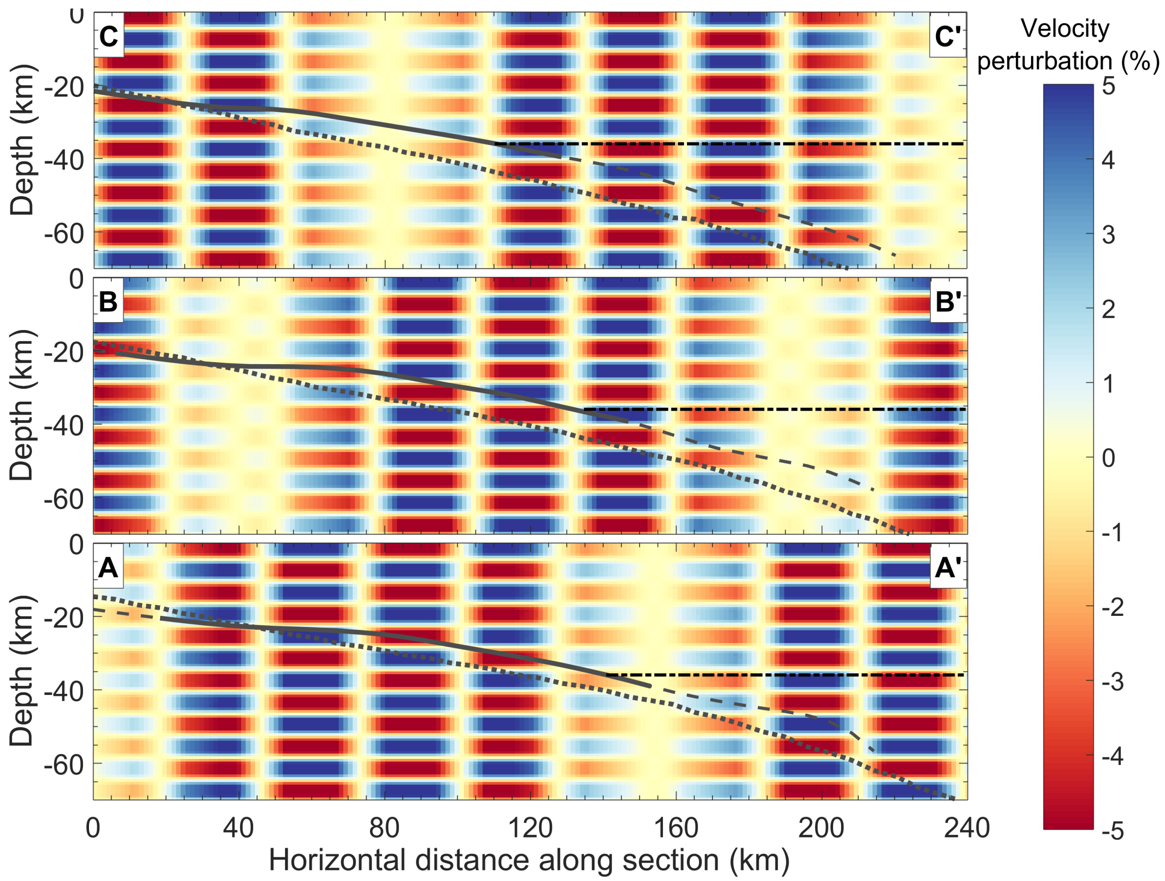

Supplementary Fig. 4: Synthetic checkerboard model. Note that profiles cross the checkerboard grid obliquely leading to some fading for certain sections

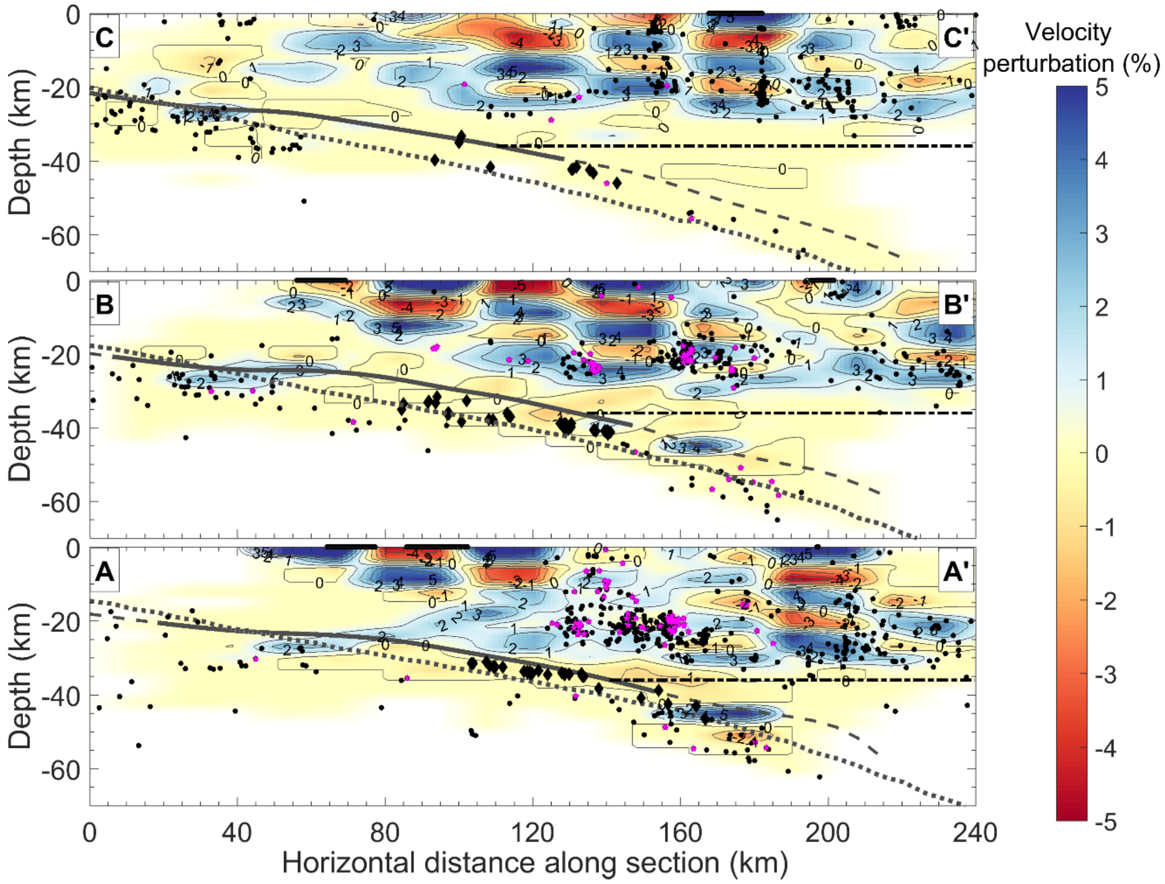

Supplementary Fig. 5: Recovered checkerboard model. Relocated seismicity in the checkerboard model is shown to illustrate data coverage.

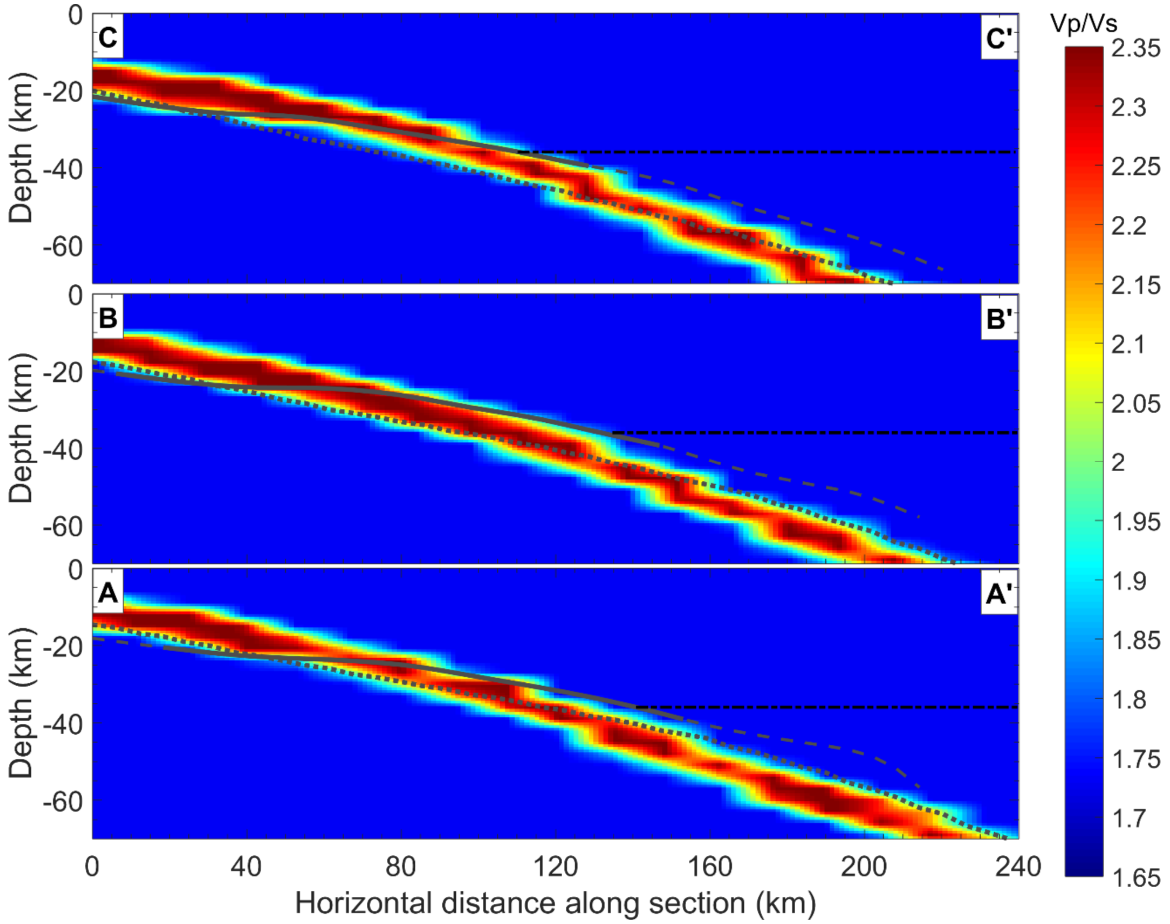

Supplementary Fig. 6: Synthetic high *V*p/*V*s LVZ model. We interpolated onto our model grid an 8 km dipping layer with a *V*p/*V*s ratio of 2.35 approximately following published slab interface models[2,3].

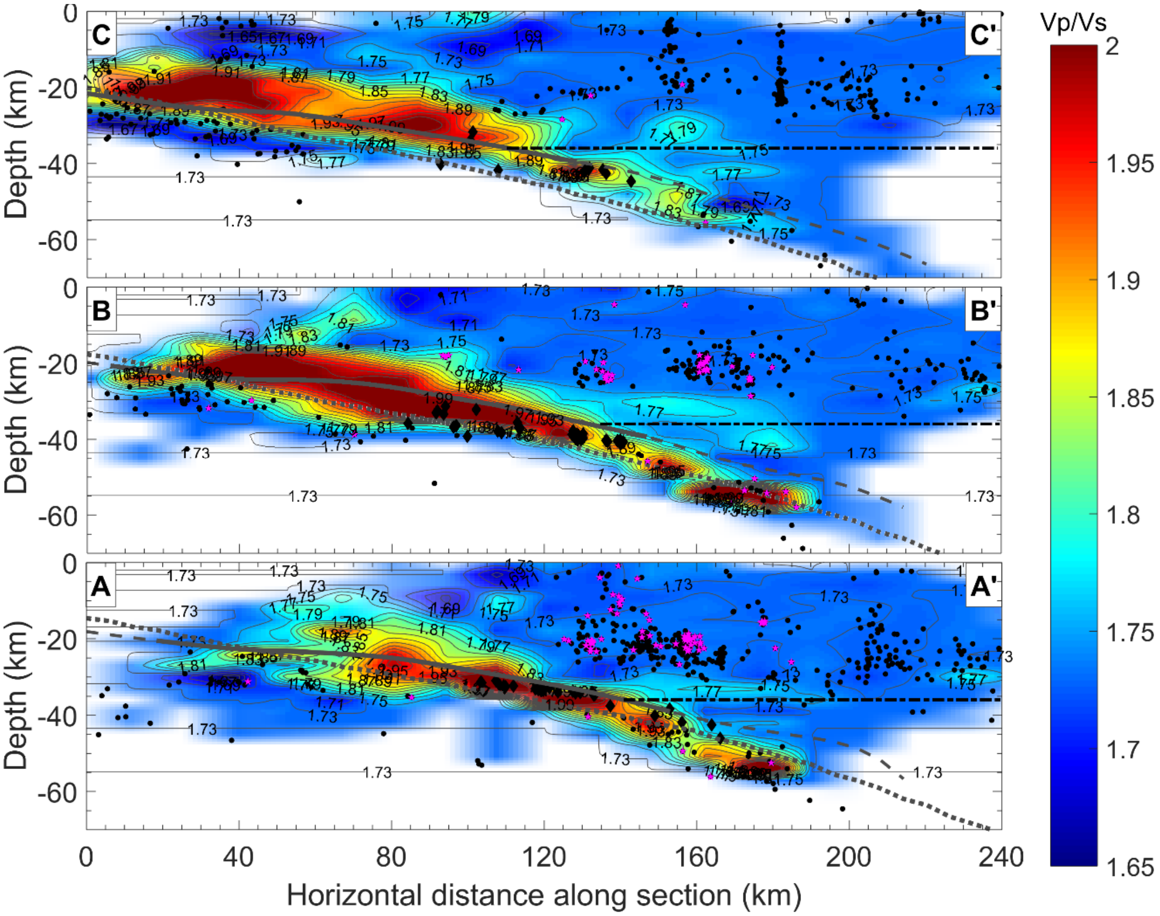

Supplementary Fig. 7: Recovered high *Vp*/*Vs* dipping layer. Relocated seismicity in the synthetic model is shown to illustrate data coverage.

Supplementary Note 2: 2D resistivity model assessment

The coordinates of the 23 magnetotelluric (MT) stations used for the resistivity inversion across southwest British Columbia are listed in Supplementary Table 1, and the same stations were used for the synthetic modelling in the model assessment.

Site	Longitude (degrees W)	Latitude (degrees N)
abc102	124.28639	48.59389
abc104	124.09167	48.58417
abc105	123.91806	48.60944
abc106	123.73333	48.64500
abc107	123.57444	48.71944
abc210	123.02500	48.47333
abc110	123.18500	48.73667
abc220	122.78611	48.64611
abc230	122.58444	48.80806
abc114	122.70667	49.01167
abc240	122.40167	48.88028
abc115	122.50667	49.01667
abc250	122.15278	48.85028
abc116	122.34917	49.00278
abc118	122.16917	49.08806
abc260	121.89222	48.89889
abc120	122.04222	49.17417
abc122	121.92583	49.24667
abc124	121.72500	49.28417
abc125	121.61389	49.31917
abc126	121.42389	49.32194
abc128	121.37556	49.36028
abc132	121.27944	49.49167

Supplementary Table 1: Coordinates of the magnetotelluric stations used in the resistivity inversion and synthetic modelling study.

Regularization parameter and L-curve analysis

A number of inversions were undertaken to explore the dependence of the final resistivity model on the parameters used to control the inversion. A key parameter to be investigated is the regularization parameter τ which controls the trade-off between overall model roughness and data misfit. Small τ values will produce resistivity models with a small data misfit but the model can be rough and contain artifacts. In contrast, larger τ values will produce smoother models that will have increased data misfit. Finding the trade-off between these competing criteria requires inversions that use a range of τ values as illustrated in Supplementary Fig. 8. The models with τ = 100 and τ = 10 are clearly too smooth and have relatively high misfits, while the model with τ = 0.1 and τ = 1 are quite rough. The optimal value of τ can be selected using the L-curve shown in Supplementary Fig. 9, where a value in the range of $\tau \sim 5$ appears to define the corner. Based on Supplementary Figs. 8 and 9, a value of τ = 5 was chosen for data analysis. For this inversion the initial root mean square (r.m.s.) misfit of 6.42 was reduced to 1.66 after 108 iterations. The data fit is illustrated in Supplementary Fig. 10 in pseudosection format.

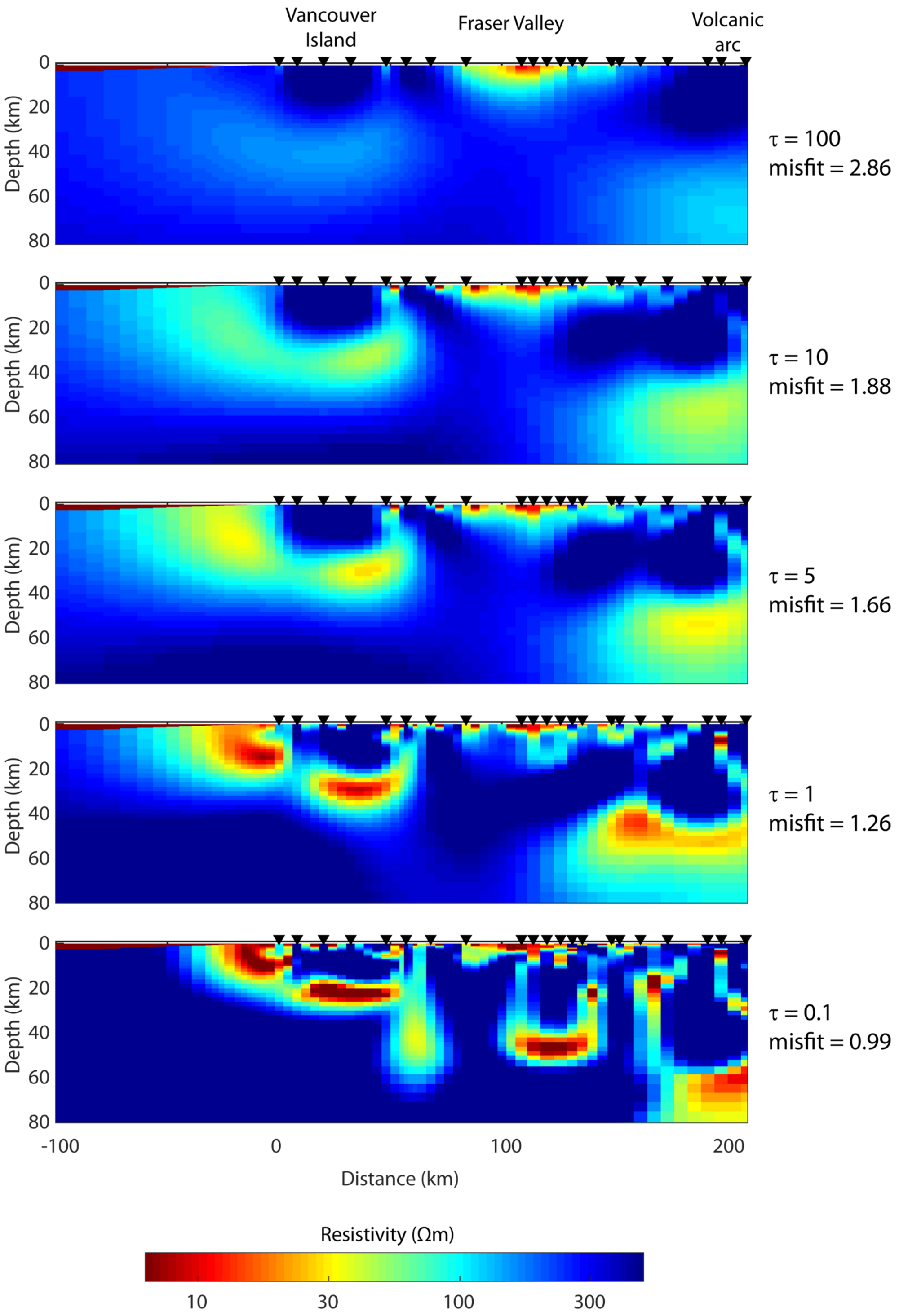

Supplementary Fig. 8: 2-D resistivity models along the ABC-S profile[4] for various trade-off parameters. MT stations denoted by the inverted black triangles. Transverse magnetic (TM) mode and vertical magnetic field transfer functions were inverted in the period range 1 – 10,000 s. The Pacific Ocean was included as a layer with resistivity 0.3 Ωm in the distance range -100 km to 0 km.

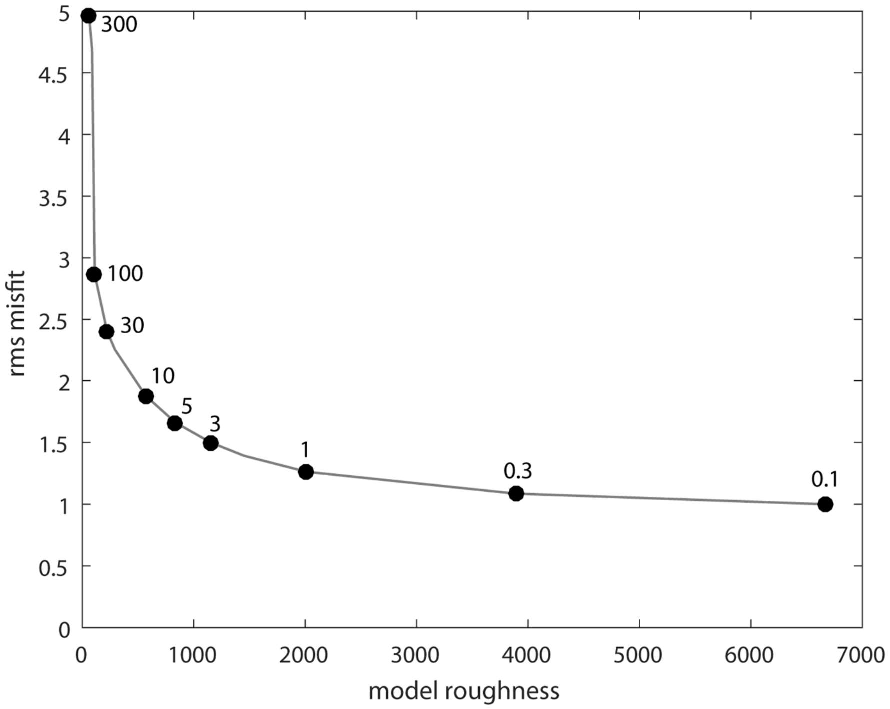

Supplementary Fig. 9: **Trade-off parameter L-curve.** Variation of final r.m.s. misfit and model roughness as the regularization parameter is varied from τ = 300 to τ = 0.1

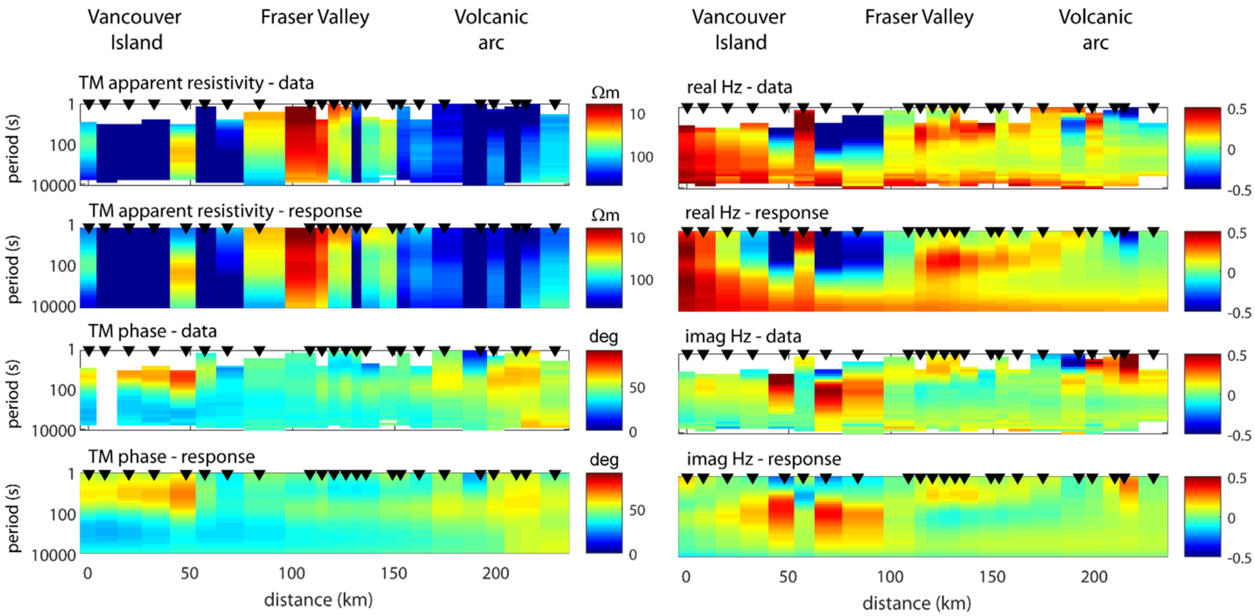

Supplementary Fig. 10: Fit of inversion response to the measured MT data for the 2-D inversion with τ= 5. Black triangles show locations of MT stations. White boxes denote data points that were removed from the analysis because they were identified as noisy or not collected.

Resolution test using synthetic inversion

Supplementary Fig. 11 shows a set of inversions used to investigate the resolution of the model corresponding to the τ=5 model in Supplementary Fig. 8. The trial models contain the following features and are based on the actual data inversion with the depth of the Juan de Fuca plate constrained by the seismological results:

- Pacific Ocean as a layer with 0.3 Ωm
- Accretionary wedge above Juan de Fuca Plate with resistivity of 20 Ωm
- Shallow sedimentary basin in Fraser Valley with resistivity of 10 Ωm
- Upper mantle conductor beneath volcanic arc with resistivity of 30 Ωm
- Dipping conductor on top of Juan de Fuca Plate with resistivity of 20 Ωm and thickness of 5 km. A range of geometries for this feature were investigated.

For each model, synthetic MT data were calculated for the actual station locations and period bands. Gaussian noise was added with noise levels of 5% in the apparent resistivity and phase and 0.06 in the vertical magnetic field transfer functions. As with the data inversions, a discontinuity was permitted at the seafloor and this avoided the low resistivity ocean layer being smoothed to unrealistic depths.

In all inversions, the model is not well resolved west of Vancouver Island because of the lack of MT stations on the seafloor. The sedimentary basin in the Fraser Valley and upper mantle conductor beneath the volcanic arc were well resolved.

The dipping conductor above the Juan de Fuca plate is well resolved, but it should be noted that the spatial smoothing imposed in the 2-D MT inversion results in a smoothed resistivity feature. This reflects the diffusive nature of MT signal propagation in the Earth; MT resolves the depth to a conductor and the conductance (depth integrated conductivity). The four examples show that the conductor is not present beneath the Fraser Valley and does not connect to the conductor beneath the volcanic arc. The upward turn beneath Straight of Georgia is clearly required by the field data.

Beneath Vancouver Island, the conductor is essentially coincident with the top of the Juan de Fuca plate. It has a thickness of 10 km and a resistivity in the range 20 - 40 Ωm. This corresponds to a conductance of 250 S. A resistivity of 30 Ωm could be explained by the presence of saline fluids in the pore space. If the fluid has a resistivity of 0.3 Ωm, mixing laws such as Archie's Law can be used to estimate the porosity. With the fluids distributed in cracks and well connected, a porosity of 1% is required. With the fluids isolated this value would be as high as 10%.

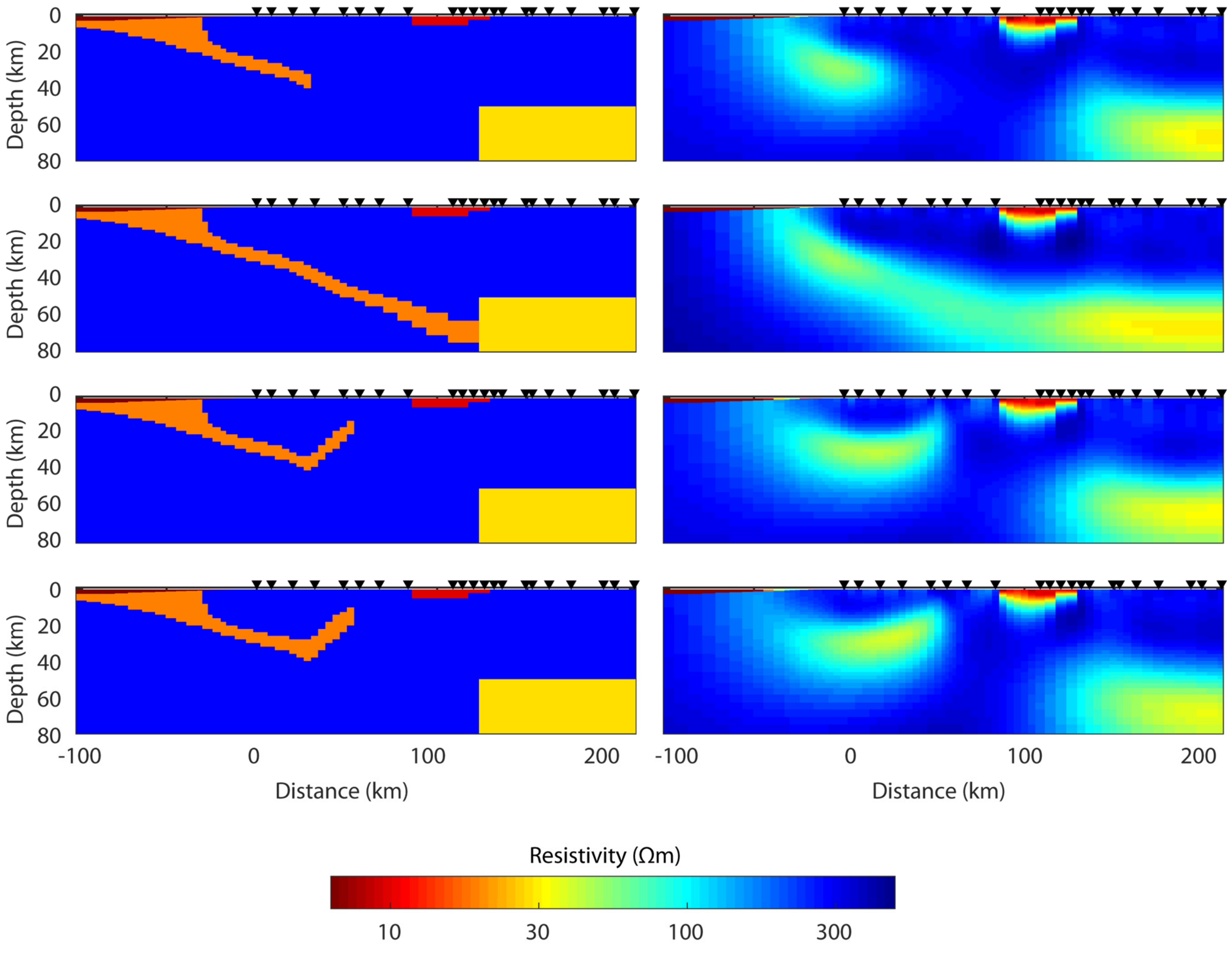

Supplementary Fig. 11: Synthetic inversion study to investigate model resolution. The left hand column shows a range of 2-D resistivity models based on the data inversion in Supplementary Fig. 8 for $\tau = 5$. Synthetic data were computed for each model and inverted using the same procedure as applied to the data inversion in Supplementary Fig.10. The right hand column shows the result of inverting the synthetic data with $\tau = 5$.

Supplementary Note 3: Migration and display of seismic reflection sections

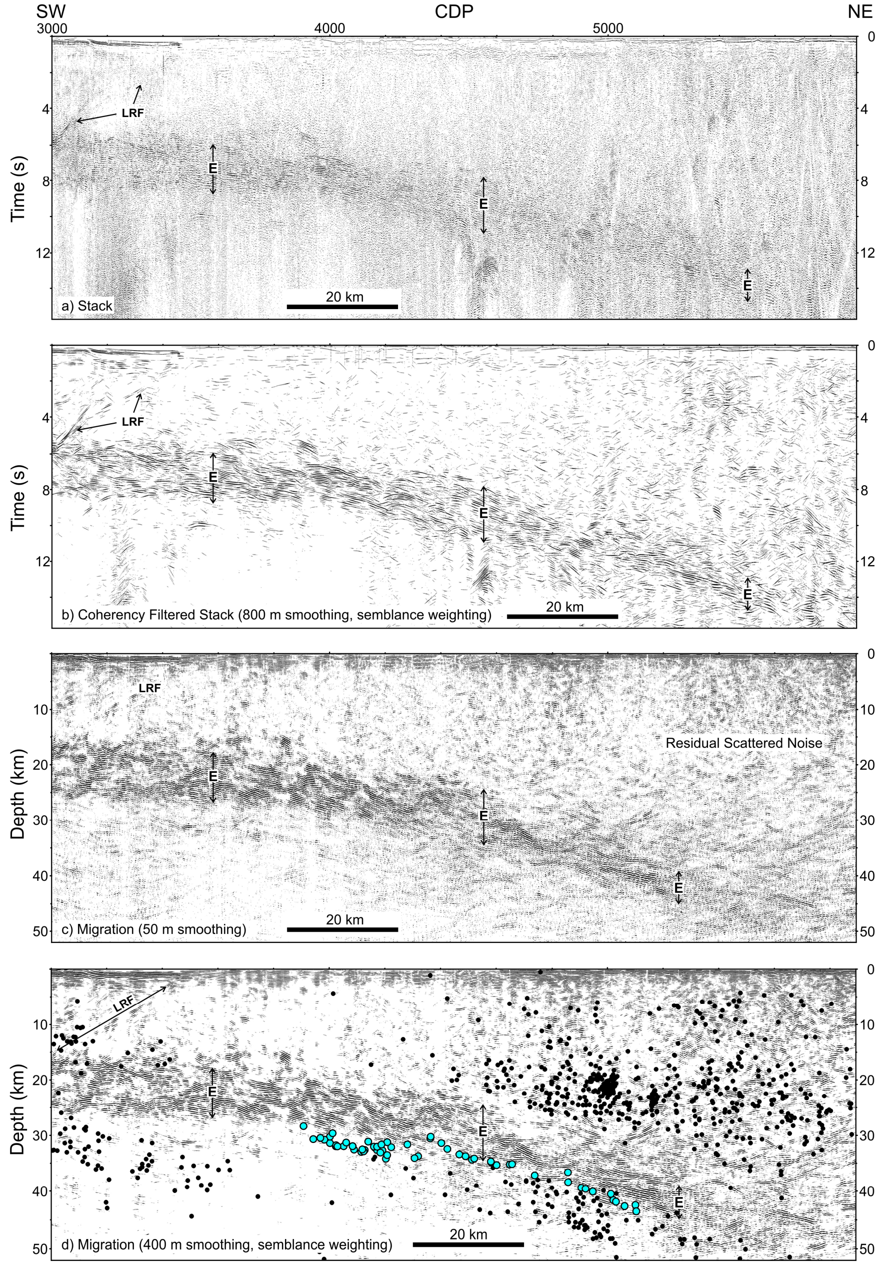

Supplementary Fig. 12: Displays of seismic reflection data around southern Vancouver Island. a) Unmigrated stack, b) Unmigrated stack with semblance weighted coherency enhancement over an 800 m long window, c) Segment migration of dips up to 25° of the unmigrated stack shown in (a); each input point is mapped to a 50 m wide output arrival, d) Segment migration of dips up to 25° of the unmigrated stack shown in (a). Each input point is mapped to a 400 m wide output arrival and also scaled by a semblance value estimated from the most coherent dip over 1600 m at the input location. Earthquakes (filled black circles) and LFEs (filled blue circles) within 20 km of the profile have been projected onto the seismic section along an azimuth of 150°. All seismic sections are displayed with trace-to-trace amplitude equalization using a 3-12 s window.

Since it is challenging to reproduce legibly long seismic sections in a compressed format the seismic sections in this paper are displayed with a threshold that removes low amplitude values. Coherency enhancement can also be applied to further improve the visibility of reflections: for unmigrated stack sections, seismic data at each sample are summed over the most coherent dip computed over a local window of traces at that location and scaled by the semblance (Supplementary Fig. 12b). To avoid the broad artifacts associated with wave-equation migration of deep seismic data, a segment migration algorithm[5], which maps each input point to an output segment of user-specified length, is employed, thus controlling the lateral smearing of the migration process, as illustrated for 50 m, i.e. no smoothing, (Supplementary Fig. 12c) and 400 m smoothing (Supplementary Fig. 12d); the input data to the migration is the stack section without coherency enhancement (Supplementary Fig. 12a).

Supplementary References

1. Savard, G., Bostock, M.G. & Christensen, N.I. Seismicity, metamorphism, and fluid evolution across the northern Cascadia fore arc. *Geochem. Geophys. Geosystems* **19**, 1881-1897 (2018).
2. Audet, P., Bostock, M.G., Boyarko, D.C., Brudzinski, M.R. & Allen, R.M. Slab morphology in the Cascadia fore arc and its relation to episodic tremor and slip. *J. Geophys. Res*. **115**, doi:10.1029/2008JB006053 (2010).
3. McCrory, P. A., Blair, J.L. & Oppenheimer, D.H. Depth to the Juan de Fuca slab beneath the Cascadia subduction margin – A 3-D model for sorting earthquakes, *U.S. Geol. Surv. Data Ser.*, 91, 1-22 (U.S. Geol. Surv., Reston, 2006).
4. Rippe, D., Unsworth, M.J. & Currie, C.A. Magnetotelluric constraints on fluid content in the upper mantle beneath the southern Cordillera: Implications for rheology. *J. Geophys. Res.* **118**, 5601-5624 (2014).
5. Calvert, A.J. A method for avoiding artifacts in the migration of deep seismic reflection data. *Tectonophysics* **388**, 201-212 (2004).
